# ∆nFGF1 Protects *β*-Cells against High Glucose-Induced Apoptosis via the AMPK/SIRT1/PGC-1*α* Axis

**DOI:** 10.1155/2022/1231970

**Published:** 2022-10-03

**Authors:** Qiong Chen, Xinwei Chen, Zhenyu Jia, Yali Du, Shujun Zhang, Wenxin Xu, Beibin Pan, Jiaxin Lou, Jianhui Zhou, Jie Zhou, Jian Sun

**Affiliations:** ^1^Pingyang Affiliated Hospital of Wenzhou Medical University, Zhejiang 325400, China; ^2^School of Pharmaceutical Sciences, Wenzhou Medical University, Zhejiang 325035, China; ^3^Department of Pediatrics, Taizhou Hospital of Zhejiang Province Affiliated to Wenzhou Medical University, Zhejiang 318000, China

## Abstract

Long-term exposure to high glucose leads to *β*-cell dysfunction and death. Fibroblast growth factor 1 (FGF1) has emerged as a promising diabetes treatment, but its pharmaceutical role and mechanism against glucolipotoxicity-induced *β*-cell dysfunction remain uncharacterized. Wild-type FGF1 (FGF1^WT^) may exhibit *in vivo* mitogenicity, but deletion of N-terminal residues 1-27 gives a nonmitogenic variant, ∆nFGF1, that does not promote cell proliferation and still retains the metabolic activity of FGF1^WT^. To investigate the roles of ∆nFGF1 on glucose regulation and potential islet *β*-cell dysfunction, *db*/*db* mice were used as a model of type 2 diabetes. The results showed that insulin secretion and apoptosis of islet *β*-cells were dramatically improved in ∆nFGF1-treated *db*/*db* mice. To further test the effects of ∆nFGF1 treatment, pancreatic *β*-cell (MIN6) cells were exposed to a mixture of palmitic acid (PA) and high glucose (HG) to mimic glucolipotoxic conditions *in vitro*. Treatment with ∆nFGF1 significantly inhibited glucolipotoxicity-induced apoptosis. Mechanistically, ∆nFGF1 exerts a protective effect on *β*-cells via activation of the AMPK/SIRT1/PGC-1*α* signaling pathway. These findings demonstrate that ∆nFGF1 protects pancreatic *β*-cells against glucolipotoxicity-induced dysfunction and apoptosis.

## 1. Introduction

Type 2 diabetes mellitus (T2DM) is characterized by persistent hyperglycemia in the context of insulin resistance [1, 2]. Insulin resistance and pancreatic *β*-cell dysfunction are considered the central characteristics of T2DM [3, 4]. Long-term high blood glucose can induce the loss of *β*-cells, considered a key step in the development of diabetes [5]. Therefore, an effective strategy to prevent diabetes mellitus may be the reduction of pathological *β*-cell apoptosis induced by high blood glucose levels.

The fibroblast growth factor (FGF) family consists of 22 members that play important roles in regulating the function of endocrine-relevant tissues and various metabolic processes [6–9]. The biological activities of FGFs are regulated by FGF receptors, and multiple FGFs may share the same FGF receptors to modulate cellular activity [10, 11]. FGF21 increases insulin secretion in diabetic islets and protects *β*-cells against apoptosis through extracellular signal-regulated kinase 1/2 (Erk1/2) and AKT pathways [12]. As a recently found metabolic modulator, FGF1 has been reported to improve insulin resistance and *β*-cell insulin secretion in diabetic mouse models [13, 14]. However, the underlying mechanisms of these effects to counter glucolipotoxicity-induced *β*-cell dysfunction remain unclear. Wild-type FGF1 (FGF1^WT^) has mitogenic activity, limiting its therapeutic potential, but a nonmitogenic FGF1 (∆nFGF1) lacking N-terminal residues 1–27 of FGF1^WT^ exhibits no apparent proliferative activity [15]. The goals of this study were to investigate the roles of ∆nFGF1 on glucose control and its protective effect against islet *β*-cell apoptosis after chronic administration in a diabetes model and determine the underlying mechanisms of these effects.

Adenosine 5′-monophosphate-activated protein kinase (AMPK) is highly expressed in metabolically active tissues and plays a crucial role in whole-body energy homeostasis [16–18]. AMPK dysregulation has been implicated in metabolic disorders like insulin resistance and T2DM [19–21]. AMPK activation may mediate the effect of FGF1 to protect against nonalcoholic fatty liver disease (NAFLD) in T2DM mice [22], and FGF4 improves blood glucose through activation of the AMPK pathway [23], implicating the AMPK pathway in the protective effects of FGF treatment on pancreatic islet *β*-cells.

In this study, we explored the glucose-lowering effects of ∆nFGF1 in insulin resistance mouse models. Our results show that ∆nFGF1 significantly inhibits *β*-cell apoptosis and improves *β*-cell function through the AMPK/SIRT1/PGC-1*α* pathway. Overall, these results indicate the therapeutic potential of ∆nFGF1 to prevent high glucose-induced *β*-cell apoptosis for T2DM treatment.

## 2. Materials and Methods

### 2.1. Cell Culture and Treatments

Mouse pancreatic *β*-cell line MIN6 (Keygen Biotech, Nanjing, China) cells were cultured in RPMI1640 medium (Gibco) accompanied with 10% fetal bovine serum (FBS, Gibco) and 1% penicillin-streptomycin. Cells were passaged every three days. Culture media was changed every 24 h.

For intracellular signaling studies, MIN6 cells were starved for 12 h followed by incubation with or without ∆nFGF1 (50 ng/mL) for 1 h. Cells were then exposed to media containing normal glucose (NG, 11.1 mM) as a control or high glucose (HG, 33 mM)+palmitic acid (PA, 0.5 mM) for 24 h and then lysed to detect protein expression by Western blot. For inhibitor experiments, MIN6 cells were also starved for 12 h and treated with AMPK inhibitor Compound C (10 *μ*M, Selleck Chemicals, S7306) or SIRT1 inhibitor EX-527 (10 *μ*M, MedChemExpress, HY-15452) or PGC-1*α* inhibitor SR-18292 (10 *μ*M, MedChemExpress, HY-101491) for 1 h and then incubated in NG or HG+PA or HG+PA+∆nFGF1 for 24 h and lysed to detect protein expression by Western blot. For siRNA knockdown experiments, MIN6 cells were seeded and grown in six-well plates for 24 h to achieve 70% confluence. Cell transfection was performed with the transfection reagent Lipofectamine 3000 in accordance with the manufacturer's instructions. A 24 h transfection of AMPK siRNA (Santa Cruz Biotechnology, sc45313) was followed by starvation and treatment as described above.

### 2.2. Cell Counting Kit-8 Assay

MIN6 cells were seeded into a 96-well plate (3000 cells/well) and serum starved in RPMI1640 medium for 24 h. The MIN6 cells were then exposed to 50 ng/mL ∆nFGF1 or FGF1^WT^ for 24 h or exposed to 33 mM HG and 0.5 mM PA in the presence or absence of 50 ng/mL ∆nFGF1 for 24 h. Cell Counting Kit-8 (CCK-8, Jiancheng Bioengineering Institute, China) was used to assess cell viability according to the manufacturer's instructions. In brief, cells treated as described above were incubated with 10 *μ*L of CCK-8 solution at 37°C for 1 h. The optical density (OD) value was detected by a microplate reader (Thermo, USA).

### 2.3. TUNEL Labeling

MIN6 cells were seeded at a total concentration of 3 × 10^5^ on a glass coverslip in six-well plates for 24 h, followed by treatment with 33 mM HG and 0.5 mM PA in the presence or absence of 50 ng/mL ∆nFGF1 for 24 h. Then, cells were washed with sterile PBS twice, fixed using 4% paraformaldehyde (PFA) for 15 min, and then permeabilized with 0.2% Triton X-100 for 5 min. Subsequently, each slide was incubated with a terminal deoxynucleotidyl transferase- (TdT-) labeled reaction mix at 37°C for 60 min in the dark. Cell nuclei were counterstained with DAPI for 10 min. Finally, fluorescent images were captured by Leica SP8 confocal microscopy using the FITC channel (Leica, Wetzlar, Germany).

### 2.4. Flow Cytometry Analysis

MIN6 cells were seeded at a total concentration of 3 × 10^5^ and cultured in six-well plates. Apoptosis of MIN6 cells was detected using an Annexin V-FITC Apoptosis Detection Kit (A211, Vazyme, Shanghai, China). In brief, treated and untreated cells were washed with cold PBS and resuspended in 500 *μ*L binding buffer and then incubated with 5 *μ*L of Propidium Iodide (PI) and 5 *μ*L of Annexin V-FITC in the dark for 10 min. The fluorescence intensity was measured using a flow cytometer (Beckman, USA) within 1 h and monitoring 1 × 10^5^ cells per sample. Apoptosis was analyzed using FlowJo VX10.

### 2.5. Animal Models

Eight-week-old male *db*/*db* (C57BLKS/J Leprdb/db) mice and normal *db*/*m* mice were purchased from GemPharmatech Co. Ltd. (Nanjing, China) and allowed to adapt to the new environment for no less than 7 days. All animals were fed with basal rodent chow and tap water *ad libitum* and housed at 22–24°C. The animal study was approved by the Institutional Animal Care and Use Committee of Wenzhou Medical University.

10-week-old *db/db* mice received every other day intraperitoneal injection of ∆nFGF1 protein (0.5 mg/kg) for eight weeks, whereas nondiabetic *db*/*m* and *db*/*db* control mice were injected with the equal amount of 0.9% saline. Body weights were recorded before injection every two days, and glucose tests were performed weekly. Random nonfasted blood glucose was measured in mouse tail venous blood using an automatic glucose monitor (Roche, Germany). Finally, mice in each group were sacrificed and the pancreatic tissues were collected for further analysis.

### 2.6. Hematoxylin and Eosin (H&E) Staining

After dehydration and hydration, tissue sections were stained with H&E (Beyotime Biotech, Nantong, China) and captured by light microscopy (Nikon, Tokyo, Japan) to evaluate histological changes. The pancreatic islet areas were measured by ImageJ (National Institutes of Health, USA).

### 2.7. Histology

For pancreatic tissue immunohistochemical (IHC) staining, tissue sections were deparaffinized, rehydrated, antigen recovered, and permeabilized with 0.2% Triton X-100 at room temperature for 15 min. After blocking with 0.5% bovine serum albumin (BSA, Sigma), sections were probed with anti-PCNA antibody (Santa, SC-25280, dilution: 1 : 100) at 4°C overnight and then incubated with anti-mouse IgG horseradish peroxidase- (HRP-) conjugated secondary antibody at room temperature for 1 h. Subsequently, sections were reacted with diaminobenzidine (DAB) for 3 min, and then, the reactions were quenched in double-distilled water, counterstained with hematoxylin, and examined by light microscopy (Nikon, Japan).

For immunofluorescence staining, samples were treated as for IHC analysis until sections were incubated with primary antibodies against insulin (Proteintech, 15848-1-AP, dilution: 1 : 100), cleaved caspase 3 (Cell Signaling, 9664, dilution: 1 : 200), SIRT1 (Proteintech, 13161, dilution: 1 : 1000), and PGC-1*α* (Proteintech, 66369, dilution: 1 : 200) overnight at 4°C. Next, the slides were incubated with goat anti-mouse (Abcam, ab150113, dilution: 1 : 1000) or goat anti-rabbit (Abcam, ab150077, dilution: 1 : 1000) antibodies for 1 h at room temperature. Sections were stained with DAPI to detect the nuclei, and the stained tissues were imaged by Leica SP8 confocal microscopy.

### 2.8. Western Blot Analysis

Briefly, 30 *μ*g of total protein extracts from MIN6 cells was separated by SDS-PAGE and transferred to PVDF membranes (Merck Millipore, IPVH00010). The primary antibodies used are as follows: phospho-AMPK (Cell Signaling, 2535S, dilution: 1 : 1000), total AMPK (Cell Signaling, 5831S, dilution: 1 : 1000), cleaved caspase 3 (Abmart, MB0711, dilution: 1 : 1000), Bax (Santa, SC-493, dilution: 1 : 1,000), Bcl-2 (Abmart, T30056, dilution: 1 : 1000), PGC-1*α* (Abmart, T56630, dilution: 1 : 1000), SIRT1 (Proteintech, 13161, dilution: 1 : 1000), and *β*-actin (Cell Signaling, 3700, dilution: 1 : 1000). Secondary antibodies (Cell Signaling, 7074 or 7076, dilution: 1 : 3000) were used to detect the immunoreactive bands at room temperature for 1 h. To visualize the immunoreactivity bands, the membranes were treated with an EasySee Western Blot Kit (Transgene Biotech, China) and densitometric analysis was carried out using ImageJ.

### 2.9. Statistical Analysis

Statistical analysis was performed using GraphPad Prism version 8.0. All data are presented as mean values ± SEM. For the comparison of two groups, unpaired Student's *t*-test (two-tailed) was performed. One-way ANOVA was used to compare more than two groups. A value of *p* < 0.05 was considered statistically significant.

## 3. Results

### 3.1. ∆nFGF1 Ameliorates Diabetes in *db*/*db* Mice

**Figure 1:**
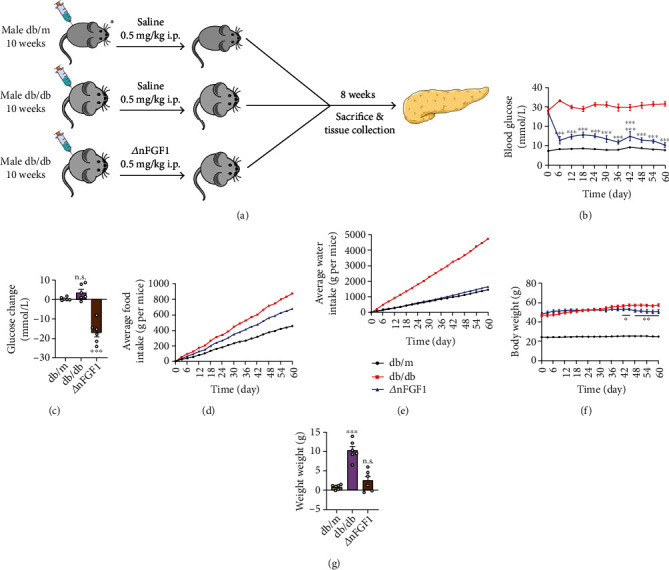
The glucose-lowering effect of ∆nFGF1 in *db*/*db* mice. (a) The 10-week-old *db/db* mice received every other day intraperitoneal injection of ∆nFGF1 (0.5 mg/kg) or saline for eight weeks, with age-matched nondiabetic *db/m* littermates served as the normal control group. (b, c) Random nonfasted blood glucose and changes, (d, e) average food intake and water intake per mouse, and (f, g) body weight alteration. All data are presented as mean values ± SEM. *n* = 6 mice per group. ^∗^*p* < 0.05, ^∗∗^*p* < 0.01, and ^∗∗∗^*p* < 0.001. n.s.: no significance.

To evaluate the protective effects of long-term treatment with ∆nFGF1 on type 2 diabetes, *db*/*db* mice received every other day intraperitoneal injection of ∆nFGF1 (0.5 mg/kg) for eight weeks (Figure 1(a)). Compared with saline-treated *db*/*m* mice, the blood glucose level was markedly increased in *db*/*db* mice from day 6 but restored to near normal level after ∆nFGF1 treatment (Figures 1(b) and 1(c)). The *db*/*db* mice treated with ∆nFGF1 exhibited effectively reduced food intake and water intake, with maintenance of a continuous upward trend in the saline-treated mice (Figures 1(d) and 1(e)), suggesting that ∆nFGF1 treatment significantly improved polydipsia and polyphagia caused by diabetes. As a consequence, the *db*/*db* mice treated with ∆nFGF1 showed notably reduced body weight compared with *db*/*db* mice treated with saline (Figures 1(f) and 1(g)). Thus, these data suggest that ∆nFGF1 suppressed body weight gain and blood glucose levels of *db*/*db* mice.

### 3.2. ∆nFGF1 Relieves *β*-Cell Apoptosis in *db*/*db* Mice

**Figure 2:**
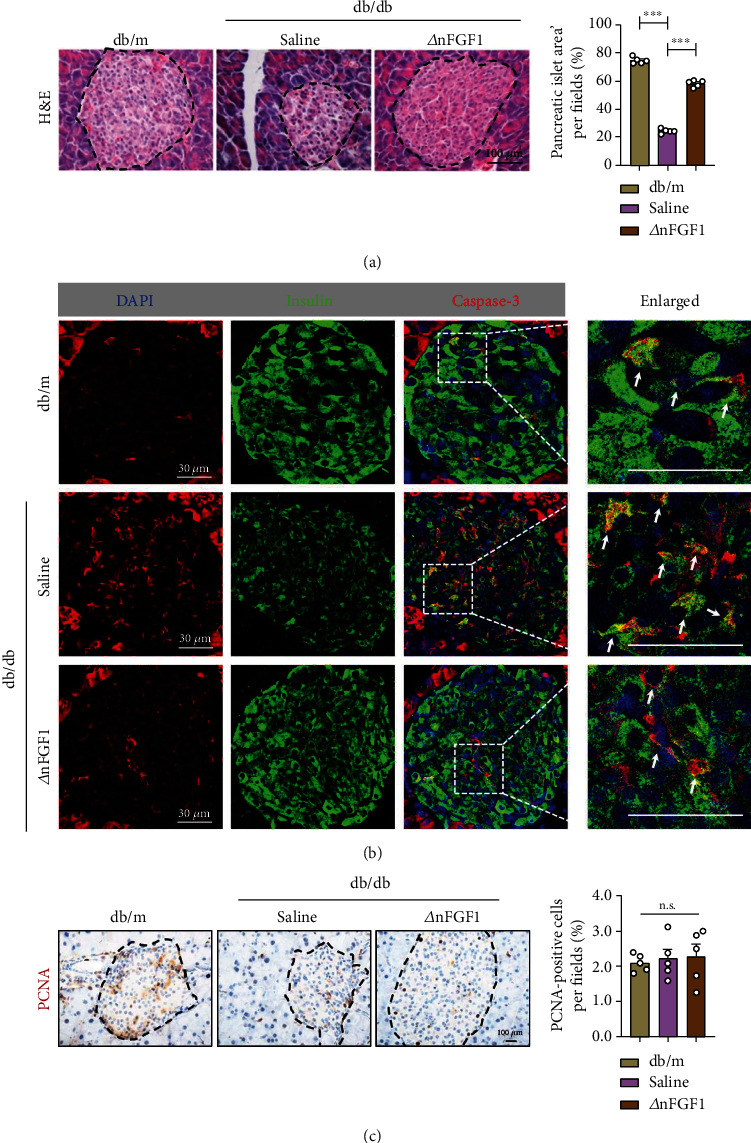
∆nFGF1 relieves *β*-cell apoptosis in *db*/*db* mice. (a) H&E staining of pancreas tissue from mice sacrificed after intraperitoneal (i.p.) injection of ∆nFGF1 or saline for 8 weeks (left panel). The black dotted lines indicate the borders of pancreatic islets. Pancreatic islet areas were quantified using ImageJ (right panel). (b) Coimmunofluorescence staining of insulin (green) and C-caspase 3 (red) in pancreatic islets. DAPI was used to stain nuclei. The white arrows indicate insulin/C-caspase 3 double-positive *β*-cells. (c) Representative PCNA-positive cells in pancreas sections (left panel). The black dotted lines indicate the borders of pancreatic islets. PCNA-positive cells were quantified using ImageJ (right panel). All data are presented as mean values ± SEM. *n* = 5 mice per group. ^∗∗∗^*p* < 0.001; n.s.: no significance.

Given that *β*-cell apoptosis is the major risk factor for diabetes, we next investigated whether ∆nFGF1 protected against pancreatic *β*-cell apoptosis. We found markedly decreased areas of pancreatic islets in *db*/*db* mice injected with saline for 8 weeks compared to that in *db*/*m* mice, with the areas significantly increased nearly to normal level in ∆nFGF1-treated *db*/*db* mice (Figure 2(a)). Caspases are crucial mediators of apoptotic process, and cleaved- (C-) caspase 3 is frequently activated cell death protease that catalyzes the specific cleavage of numerous crucial cellular proteins [24, 25]. To further investigate whether ∆nFGF1 countered *β*-cell apoptosis in *db*/*db* mice, we performed coimmunostaining of insulin and C-caspase 3 in pancreatic islets. Compared with the saline-treated *db*/*m* mice, the numbers of insulin/C-caspase 3 double-positive *β*-cells were significantly increased in the saline-treated *db*/*db* mice. Conversely, ∆nFGF1 treatment notably decreased the numbers of double-positive pancreatic *β*-cells (Figure 2(b)). Additionally, there was no significant increase in immunostaining for proliferating cell nuclear antigen (PCNA) in pancreatic islets of *db*/*db* mice treated with ∆nFGF1 compared with that of *db*/*db* mice treated with saline (Figure 2(c)). Collectively, these data suggest that ∆nFGF1 protects against diabetes by inhibiting *β*-cell apoptosis without affecting cell proliferation in *db*/*db* mice.

### 3.3. ∆nFGF1 Protects MIN6 Cells against Glucolipotoxicity-Induced Apoptosis

**Figure 3:**
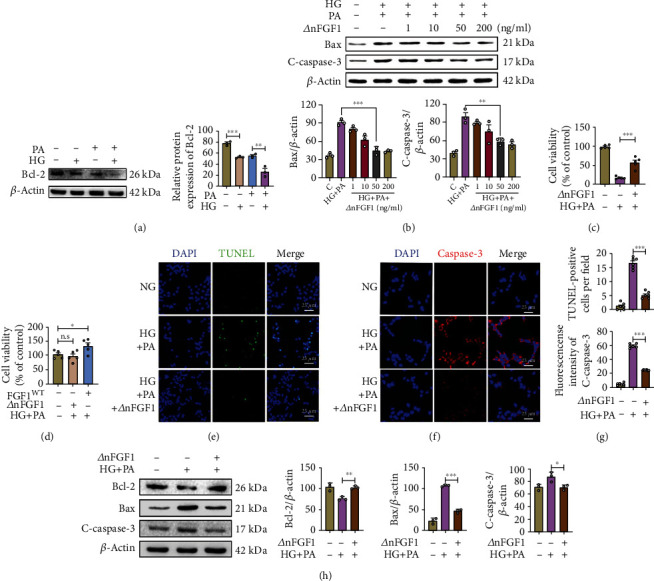
∆nFGF1 inhibits apoptosis of MIN6 cells. MIN6 cells were exposed to NG (11.1 mM) and HG+PA (33 mM HG+0.5 mM PA) in the presence or absence of ∆nFGF1 for 24 h. MIN6 cells were exposed for 24 h to HG or PA. (a) The expression of Bcl-2 was analyzed by Western blot (left panel) and quantified using ImageJ (right panel). (b) The expression of Bax and cleaved- (C-) caspase 3 in MIN6 cells under different concentrations of ∆nFGF1 was analyzed by Western blot (upper panel) and quantitated using ImageJ (lower panels). (c) The survival rate of MIN6 cells for above treatments. (d) The proliferation of MIN6 cells after treatment with FGF1^WT^ and ∆nFGF1. Representative immunofluorescence images of MIN6 cells stained with (e) TUNEL (green) and (f) C-caspase 3 (red). DAPI was used to stain nuclei. (g) The numbers of TUNEL-positive cells (upper panel) and the fluorescence intensity of C-caspase 3 were quantified using ImageJ (lower panel). (h) Bax, Bcl-2, and C-caspase 3 expression was analyzed by Western blot (left panel) and quantitated by ImageJ (right panels). *β*-Actin was used as a control. All data are presented as mean values ± SEM. NG: normal glucose; HG: high glucose; PA: palmitic acid. ^∗^*p* < 0.05, ^∗∗^*p* < 0.01, and ^∗∗∗^*p* < 0.001. n.s.: no significance.

Long-term hyperglycemia and high levels of circulating free fatty acids (FFAs) are related to pancreatic *β*-cell dysfunction [26]. To further evaluate the effect of ∆nFGF1, we next explored the damage effects of hyperglycemia and/or hyperlipidemia on MIN6 cells. Exposure to both 33 mM high glucose (HG) and 0.5 mM palmitic acid (PA) resulted in a notable decrease in antiapoptotic protein Bcl-2 expression compared with the untreated group (Figure 3(a)). Based on the established cell apoptosis model, we asked if ∆nFGF1 could inhibit cell apoptosis. Western blot analysis showed that the expression levels of proapoptotic protein C-caspase 3 and Bax significantly decreased in a dose-depended manner in ∆nFGF1-treated MIN6 cells, with an optimal concentration of ∆nFGF1 at 50 mg/mL (Figure 3(b)).

To mimic *in vivo* hyperglycemia and hyperlipidemia conditions, MIN6 cells were treated with 0.5 mM PA and 33 mM HG for 24 h and cell viability was detected by CCK-8 assays. As shown in Figure 3(c), HG and PA combined treatment significantly decreased cell viability and ∆nFGF1 treatment notably increased cell survival. Importantly, compared with the increased cell proliferation in the wild-type FGF1- (FGF1^WT^-) treated group, ∆nFGF1 treatment showed no effect on cell proliferation (Figure 3(d)). To confirm this effect of ∆nFGF1 was due to cell apoptosis, we next performed TUNEL assay to directly examine cell apoptosis. We found that ∆nFGF1 alleviated HG+PA-induced apoptosis of MIN6 cells as evidenced by decreased numbers of TUNEL-positive cells (Figures 3(e) and 3(g)). In parallel, immunofluorescence staining showed that ∆nFGF1 treatment markedly reduced C-caspase 3 expression induced by glucolipotoxicity in MIN6 cells (Figures 3(f) and 3(g)). Consistent with the immunostaining results, Western blot analysis revealed that treatment of ∆nFGF1 notably reversed the HG+PA-induced increased Bax and C-caspase 3 expression and decreased Bcl-2 (Figure 3(h)). Taken together, these results demonstrate that ∆nFGF1 can effectively inhibit glucolipotoxicity-induced apoptosis in MIN6 cells.

### 3.4. ∆nFGF1 Induces the Activation of the AMPK/SIRT1/PGC-1*α* Signaling Pathway

**Figure 4:**
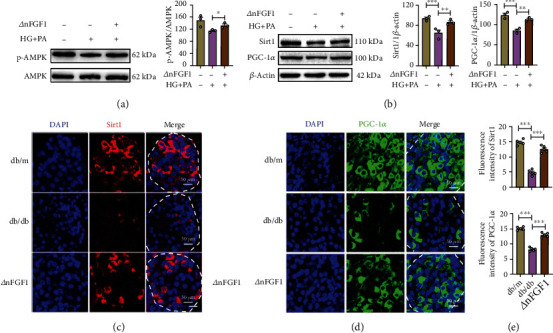
∆nFGF1 induces activation of AMPK/Sitr1/PGC-1*α* signaling. MIN6 cells were exposed to NG (11.1 mM) and HG+PA (33 mM HG+0.5 mM PA) in the absence or presence of ∆nFGF1 for 24 h. (a) The expression of phosphorylated- (p-) AMPK and AMPK analyzed by Western blot (left panel) and quantified by ImageJ (right panel) (*n* = 3). (b) The expression levels of SIRT1 and PGC-1*α* analyzed by Western blot (left panel) and quantified by ImageJ (right panel) (*n* = 3). *β*-Actin was used as a loading control. (c, d) Immunostaining for SIRT1 (red) and PGC-1*α* (green) in paraffin-embedded islets. DAPI was used to stain cell nuclei. *n* = 5 mice per group. (e) Quantitative analysis of fluorescence intensity of SIRT1 (upper panel) and PGC-1*α* (lower panel). All data are presented as mean values ± SEM. ^∗^*p* < 0.05, ^∗∗^*p* < 0.01, and ^∗∗∗^*p* < 0.001.

Numerous studies have demonstrated that FGF1 can activate the AMPK signaling pathway, which plays important pleiotropic roles in cellular responses to metabolic stress [27]. Activated AMPK further stimulates SIRT1 and subsequently increases the PGC-1*α* expression, thus playing a central regulatory role in energy metabolism [28]. We next evaluated the AMPK/SIRT1/PGC-1*α* signaling pathway in MIN6 cells after ∆nFGF1 treatment. We found that ∆nFGF1 significantly increased AMPK phosphorylation in HG+PA-induced MIN6 cells (Figure 4(a)). The ∆nFGF1 treatment group also exhibited increased expression of SIRT1 and PGC-1*α* (Figure 4(b)). Further, we examined the expression of SIRT1 and PGC-1*α* in pancreatic tissues and found higher protein levels of SIRT1 and PGC-1*α* in islets of ∆nFGF1-treated *db*/*db* mice than of *db*/*db* control mice (Figures 4(c)–4(e)). Therefore, we speculate that ∆nFGF1 modulates the AMPK/SIRT1/PGC-1*α* axis.

### 3.5. ∆nFGF1 Inhibits Glucolipotoxicity-Induced Apoptosis in MIN6 Cells via AMPK/SIRT1/PGC-1*α* Signaling

**Figure 5:**
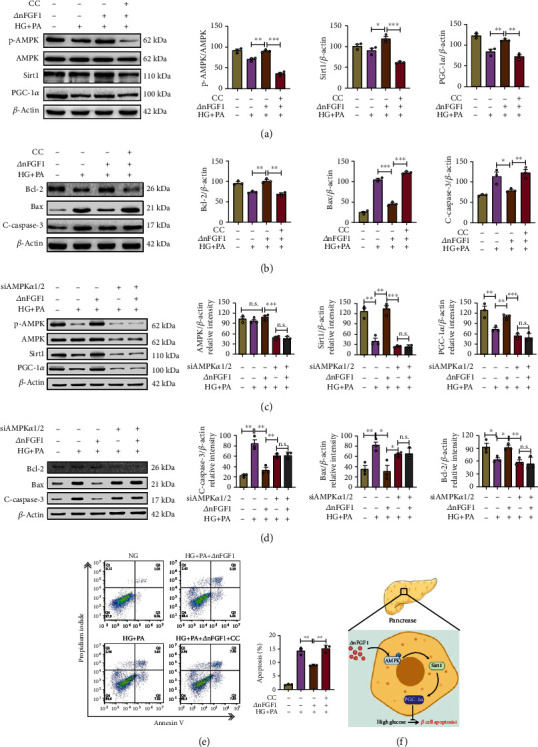
∆nFGF1 inhibits glucolipotoxicity-induced apoptosis in MIN6 cells via AMPK/SIRT1/PGC-1*α* signaling. MIN6 cells were exposed to NG (11.1 mM) and HG+PA (33 mM HG+0.5 mM PA) with or without the AMPK inhibitor Compound C (CC) in the presence or absence of ∆nFGF1 for 24 h. (a) The protein levels of p-AMPK, SIRT1, and PGC-1*α* analyzed by Western blot (left panel) and quantified by ImageJ (right panels) (*n* = 3). (b) The protein expression levels of Bcl-2, Bax, and C-caspase 3 analyzed by Western blot (left panel) and quantified by ImageJ (right panels) (*n* = 3). (c) MIN6 cells were transfected with AMPK siRNA for 48 h before exposure to NG (11.1 mM) and HG+PA (33 mM HG+0.5 mM PA) in the presence or absence of ∆nFGF1 for 24 h. The protein expression levels of p-AMPK, SIRT1, and PGC-1*α* analyzed by Western blot (left panel) and quantified by ImageJ (right panels) (*n* = 3). (d) The protein expression levels of Bcl-2, Bax, and C-caspase 3 analyzed by Western blot (left panel) and quantified by ImageJ (right panels) (*n* = 3). *β*-Actin was used as a loading control. (e) Apoptosis of *β*-cells in each group was evaluated by flow cytometry analysis. The apoptotic rate was calculated as the percentage of Annexin V-positive cells divided by the total number of cells. (f) Schematic diagram illustrating the model of ∆nFGF1-mediated inhibition of pancreatic *β*-cell apoptosis in T2DM. All data are presented as mean values ± SEM. ^∗^*p* < 0.05, ^∗∗^*p* < 0.01, and ^∗∗∗^*p* < 0.001. n.s.: no significance.

To determine whether the inhibitory effect of ∆nFGF1 on cell apoptosis resulted from ∆nFGF1-induced activated AMPK signaling, we used the AMPK inhibitor Compound C (CC) to inhibit AMPK activity of MIN6 cells. Western blot result revealed that ∆nFGF1 increased the expression of p-AMPK, SIRT1, and PGC-1*α*, while CC abolished the ∆nFGF1-induced expression increase of these proteins (Figure 5(a)). Next, we investigated the effect of AMPK inhibition on apoptosis of islet *β*-cells and found that ∆nFGF1 treatment significantly increased the antiapoptotic protein expression of Bcl-2 and decreased the proapoptotic protein expression of Bax and C-caspase 3. Conversely, the antiapoptotic effects induced by ∆nFGF1 were impeded by CC treatment (Figure 5(b)). To further confirm that ∆nFGF1 inhibits cell apoptosis through AMPK signaling, we used siRNA to knockdown AMPK expression in MIN6 cells. We found that AMPK siRNA treatment notably inhibited the activation of SIRT1 and PGC-1*α* signaling by ∆nFGF1 (Figure 5(c)). In parallel, the antiapoptotic signaling induced by ∆nFGF1 was inhibited by AMPK siRNA (Figure 5(d)). Moreover, flow cytometry assay showed that ∆nFGF1 significantly alleviated glucolipotoxicity-induced apoptosis of cells, whereas cotreatment with AMPK inhibitor CC counteracted this effect of ∆nFGF1 (Figure 5(e)), suggesting the importance of AMPK in the regulation of apoptosis in MIN6 cell.

To confirm that ∆nFGF1 protects *β*-cell apoptosis by activating AMPK/SIRT1/PGC-1*α* signaling, we used EX-527 (SIRT1 inhibitor) and SR-18292 (PGC-1*α* inhibitor) to inhibit the activities of SIRT1 and PGC-1*α*, respectively. As expected, Western blot results showed that EX-527 abolished the increased expression of SIRT1 and PGC-1*α* and antiapoptotic effects induced by ∆nFGF1 (Supplementary Figure S1A). Similar results were also obtained after cotreatment with SR-18292 (Supplementary Figure S1B). All these results suggest that ∆nFGF1 inhibits apoptosis of MIN6 cells through activating the AMPK/SIRT1/PGC-1*α* signaling pathway.

## 4. Discussion

Stable *β*-cell numbers are critical in insulin resistance and secretion, with significant reductions in *β*-cell functions in T2DM [29–31]. In this study, we determined that ∆nFGF1, a nonmitogenic truncation of FGF1, effectively reduces blood glucose in T2DM mice. More importantly, ∆nFGF1 can protect *β*-cells from apoptosis induced by high glucose levels through AMPK/SIRT1/PGC-1*α* signaling *in vivo* and *in vitro*.

Administration of ∆nFGF1 to *db*/*db* mice resulted in a notable decrease of blood glucose. Additionally, insulin secretion and apoptosis of islet *β*-cells were dramatically improved in ∆nFGF1-treated *db*/*db* mice. Immunofluorescence staining revealed significant more insulin-positive pancreatic *β*-cells in the ∆nFGF1-treated *db*/*db* mice compared with that in the *db*/*db* control mice. Interestingly, ∆nFGF1 treatment of *db/db* mice for 8 weeks did not increase the number of PCNA-positive islet cells. As a crucial cellular energy sensor, AMPK regulates energy metabolism and promotes glucose uptake through the modulation of metabolic cell signaling molecules [32–34]. Previous studies identified the activation of the AMPK/SIRT1 pathway as a potential target to inhibit apoptosis in diabetic mouse models [35, 36]. We tested the effects of ∆nFGF1 treatment on this pathway using MIN6, a pancreatic *β*-cell line, and found that treatment with ∆nFGF1 significantly inhibited glucolipotoxicity-induced apoptosis, and consistent with previous reports, we observed a reduction in phosphorylated- (p-) AMPK and Sirtuin-1 (SIRT1) expression.

We demonstrated that ∆nFGF1 can attenuate glucolipotoxicity-induced apoptosis of MIN6 cells via activation of the AMPK/SIRT1 signaling pathway, indicating that ∆nFGF1 promotes SIRT1 signaling via AMPK activation. AMPK can promote mitochondrial synthesis via the direct phosphorylation of PGC-1*α* [37]. SIRT1 also acts upstream of PGC-1*α* [38]. Our results showed that ∆nFGF1 acts to phosphorylate AMPK and increase SIRT1 and PGC-1*α* expression. We also found that ∆nFGF1 can directly inhibit proapoptotic protein C-caspase 3 and Bax as well as increase antiapoptotic protein Bcl-2 in HG+PA-treated MIN6 cells. Further, the use of an AMPK inhibitor, Compound C, abolished the protective effects of ∆nFGF1. Overall, our results revealed that ∆nFGF1 induces AMPK phosphorylation to increase SIRT1 and PGC-1*α* expression and inhibit apoptosis in pancreatic *β*-cells (Figure 5(f)).

In summary, this study uncovered a crucial role of ∆nFGF1 to inhibit *β*-cell apoptosis and promote cell survival in diabetes. Long-term application of ∆nFGF1 significantly reduced circulating blood glucose and increased the number of *β*-cells in *db*/*db* mice, by promoting insulin biosynthesis and increasing *β*-cell survival. Further investigation of the mechanism revealed that ∆nFGF1 inhibits *β*-cell apoptosis via activating AMPK/SIRT1/PGC-1*α* signaling to improve *β*-cell function. Our results illustrate the effectiveness of ∆nFGF1 as a potential treatment for high glucose-induced *β*-cell apoptosis and T2DM.

## Data Availability

The data used to support the findings of this study are included within the article.

## Supplementary Materials

Supplementary Materials Figure S1: inhibition of the activity of SIRT1 and PGC-1*α* blocks the protective effects of ∆nFGF1 on *β*-cell apoptosis. MIN6 cells were exposed to NG (11.1 mM) and HG+PA (33 mM HG+0.5 mM PA) in the presence or absence of ∆nFGF1 with or without 10 *μ*M EX-527 (Sirt1 inhibitor) and 10 *μ*M SR-18292 (PGC-1*α* inhibitor) for 24 h. (A) The protein expression of Sirt1, PGC-1*α*, Bcl-2, Bax, and C-caspase 3 analyzed by Western blot (left panel) and quantitated using ImageJ (right panels) (*n* = 3). (B) The protein expression of PGC-1*α*, Bcl-2, Bax, and C-caspase 3 analyzed by Western blot (left panel) and quantitated using ImageJ (right panels) (*n* = 3). All data are presented as mean ± SEM. NG: normal glucose; HG: high glucose; PA: palmitic acid. ^∗^*p* < 0.05, ^∗∗^*p* < 0.01, and ^∗∗∗^*p* < 0.001. n.s.: no significance.
