# Supplementary material for: ∆nFGF1 Protects β-Cells against High Glucose-Induced Apoptosis via the AMPK/SIRT1/PGC-1α Axis
Source: Oxid Med Cell Longev. 2022 Oct 3;2022:1231970. doi: 10.1155/2022/1231970 (PMC9550415; doi:10.1155/2022/1231970)
Supplement: Supplementary Materials — Figure S1: inhibition of the activity of SIRT1 and PGC-1α blocks the protective effects of ∆nFGF1 on β-cell apoptosis. MIN6 cells were exposed to NG (11.1 mM) and HG+PA (33 mM HG+0.5 mM PA) in the presence or absence of ∆nFGF1 with or without 10 μM EX-527 (Sirt1 inhibitor) and 10 μM SR-18292 (PGC-1α inhibitor) for 24 h. (A) The protein expression of Sirt1, PGC-1α, Bcl-2, Bax, and C-caspase 3 analyzed by Western blot (left panel) and quantitated using ImageJ (right panels) (n = 3). (B) The protein expression of PGC-1α, Bcl-2, Bax, and C-caspase 3 analyzed by Western blot (left panel) and quantitated using ImageJ (right panels) (n = 3). All data are presented as mean ± SEM. NG: normal glucose; HG: high glucose; PA: palmitic acid. ∗p < 0.05, ∗∗p < 0.01, and ∗∗∗p < 0.001. n.s.: no significance. [file 1231970.f1.docx]

# Supplementary Materials for

**∆nFGF1 protects β-cells against high glucose-induced apoptosis via AMPK/SIRT1/PGC-1alpha axis**

Qiong Chen^1,2,†^, Xinwei Chen^2,†^, Zhenyu Jia^2,†^, Yali Du^2^, Shujun Zhang^2^, Wenxin Xu^2^, Beibin Pan^2^, Jiaxin Lou^2^, Jianhui Zhou^3,*^, Jie Zhou^1,*^, Jian Sun^1,2,*^

^†^These authors have contributed equally to this work.

**^*^Correspondence:**

Jian Sun (sunjian@wmu.edu.cn)

Jie Zhou (zhoujie.0628@163.com)

Jianhui Zhou (zhoujh5902@enzemed.com)


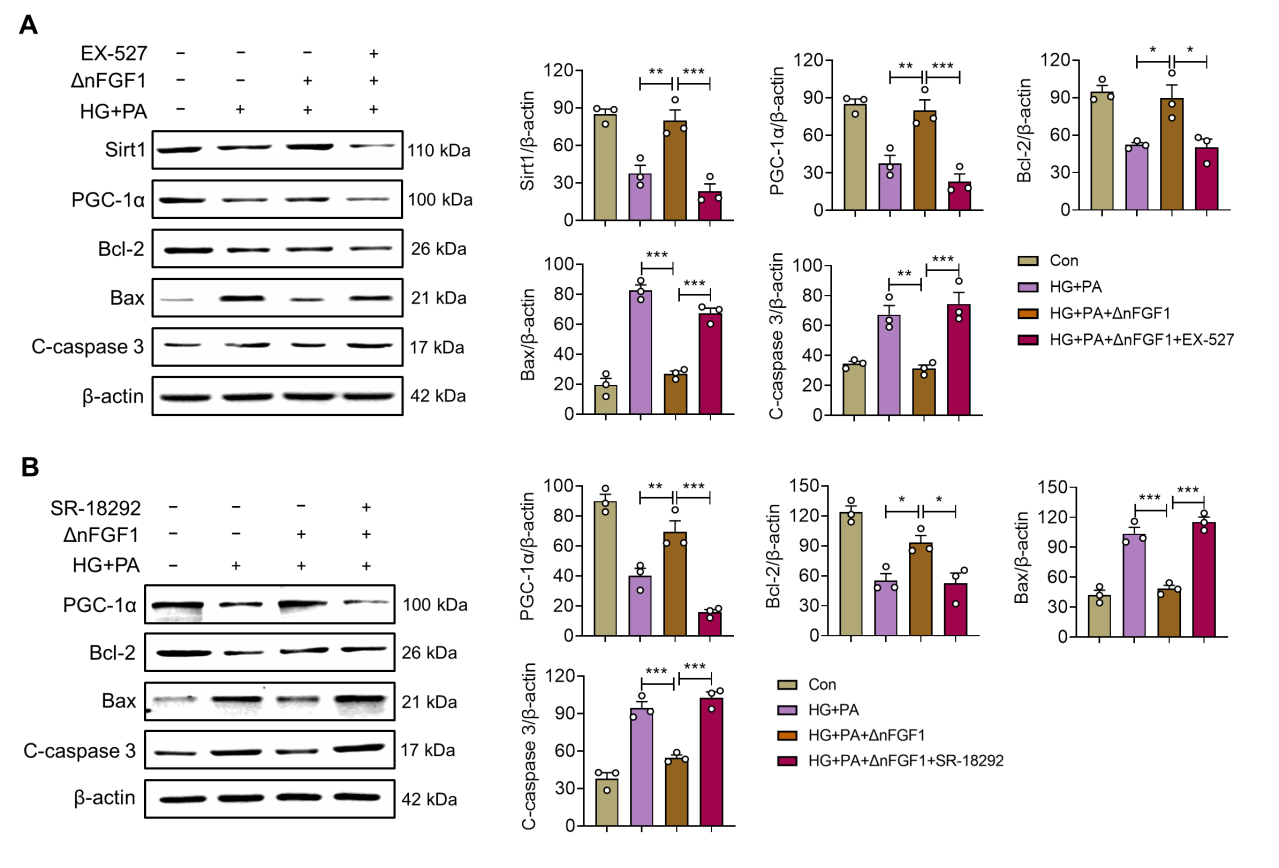


**Fig. S1** **Inhibition of the activity of SIRT1 and PGC-1α blocks the protective effects of ∆nFGF1 on β-cell apoptosis.** MIN 6 cells were exposed to NG (11.1 mM) and HG+PA (33 mM HG+0.5 mM PA) in the presence or absence of ∆nFGF1 with or without 10 μM EX-527 (Sirt1 inhibitor) and 10 μM SR-18292 (PGC-1α inhibitor) for 24 h. **(A)** The protein expression of Sirt1, PGC-1α, Bcl-2, Bax, and C-caspase 3 analyzed by Western blot (left panel) and quantitated using ImageJ (right panels) (n = 3). **(B)** The protein expression of PGC-1α, Bcl-2, Bax, and C-caspase 3 analyzed by Western blot (left panel) and quantitated using ImageJ (right panels) (n = 3). All data are presented as mean ± SEM. NG: normal glucose; HG: high glucose; PA: palmitic acid. ^*^p < 0.05, ^**^p < 0.01 and ^***^p < 0.001. n.s.: no significance.
